# Editorial for the Special Issue “Squamous Cell Cancer of the Head and Neck—Time to Arrive in the 21st Century of Oncology”

**DOI:** 10.3390/ijms22041592

**Published:** 2021-02-05

**Authors:** Sven Perner, Christian Idel

**Affiliations:** 1 Institute of Pathology, University of Luebeck and University Hospital Schleswig-Holstein, Campus Luebeck, Ratzeburger Allee 160, 23538 Luebeck, Germany; 2 Pathology, Research Center Borstel, Leibniz Lung Center, Parkallee 1-40, 23845 Borstel, Germany; 3 Department of Otorhinolaryngology, University of Luebeck, Ratzeburger Allee 160, 23538 Luebeck, Germany; Christian.Idel@uksh.de

Squamous cell carcinoma of the head and neck (HNSCC) is the sixth most common cancer worldwide. Despite enormous research efforts over the past decades, the prognosis is still rather poor. In non-human papillomavirus (HPV)-related (p16 negative) tumors, the 5-year survival rate is still only 40–60% over all tumor stages. In advanced stages, the 2-year survival is even less, since 30–50% of patients develop local or regional recurrence.

So far, the most common therapeutic options for primary HNSCC and their lymph node metastases are still surgery and/or chemoradiotherapy. However, these therapies often go hand in hand with severe side effects that are hard for patients to endure. 

We are receiving more and more evidence that the heterogeneous group of HNSCC probably needs a clearer division into biological and molecular subgroups. Tumors originate from different locations and reflect different tumor biology. The most prominent difference is found in oropharyngeal cancers in which the human papillomavirus (HPV) has a huge impact on the overall survival of patients. Additionally, HNSCC of different sites of origin differs in their response to irradiation since primary tumors of the hypopharynx have the worst response towards radiotherapy. Furthermore, the recent introduction of immune therapies for solid cancers with the use of immune checkpoint inhibitors (ICI) has increased the overall survival rates of many patients, regardless of their cancer type. The most beneficial impact was observed in malignant melanoma therapy. Therefore, high hopes were set for the treatment of recurrent HNSCC. The results of several phase-III clinical trials showed a significant improvement compared to the standard of care, in particular in patients with a higher PD-L1 expression. While new treatment regimens are being introduced in several cancer entities, the treatment of HNSCC still remains far behind. In particular, the introduction of driver mutation-associated therapies—e.g., with tyrosine kinase inhibitors (TKI)—is also promising. Additionally, PIK3CA inhibitor showed promising first results in HNSCC with an activating PIK3CA mutation. 

All these factors show that we still need a better understanding of HNSCC to obtain better treatment options. With this Special Issue, we aim to offer a platform to improve our molecular understanding of HNSCC to finally move their treatment into the 21st century.

